# The rootstock shape microbial diversity and functionality in the rhizosphere of *Vitis vinifera* L. cultivar Falanghina

**DOI:** 10.3389/fpls.2023.1205451

**Published:** 2023-08-14

**Authors:** Daniela Zuzolo, Maria Antonietta Ranauda, Maria Maisto, Maria Tartaglia, Antonello Prigioniero, Alessandra Falzarano, Giuseppe Marotta, Rosaria Sciarrillo, Carmine Guarino

**Affiliations:** ^1^ Department of Science and Technologies, University of Sannio, Benevento, Italy; ^2^ Department of Law, Economics, Management and Quantitative Methods (DEMM), University of Sannio, Benevento, Italy

**Keywords:** rootstock, grapevine, microbial terroir, rhizosphere, holobiont, microbial diversity

## Abstract

The rhizosphere effect occurring at the root-soil interface has increasingly been shown to play a key role in plant fitness and soil functionality, influencing plants resilience. Here, for the first time, we investigated whether the rootstock genotype on which *Vitis vinifera* L. cultivar Falanghina is grafted can influence the rhizosphere microbiome. Specifically, we evaluated to which extent the 5BB and 1103P rootstocks are able to shape microbial diversity of rhizosphere environment. Moreover, we explored the potential function of microbial community and its shift under plant genotype influence. We investigated seven vineyards subjected to the same pedo-climatic conditions, similar age, training system and management and collected twelve rhizosphere soil samples for metagenomic analyses and composite soil samples for physical-chemical properties. In this study, we used 16S rRNA gene-based metagenomic analysis to investigate the rhizosphere bacterial diversity and composition. Liner discriminant analysis effect size (LEFSe) was conducted for metagenomic biomarker discovery. The functional composition of sampled communities was determined using PICRUSt, which is based on marker gene sequencing profiles. Soil analyses involved the determination of texture, pH, Cation Exchange Capacity (CSC), Organic Carbon (OC), electrical conductivity (EC), calcium (Ca), magnesium (Mg), potassium (K) content, Phosphorous (P), nitrogen (N). The latter revealed that soil features were quite homogenous. The metagenomic data showed that the bacterial alpha-diversity (Observed OTUs) significantly increased in 1103P rhizosphere microbiota. Irrespective of cultivar, Pseudomonadota was the dominant phylum, followed by Actinomycetota > Bacteroidota > Thermoproteota. However, Actinomycetota was the major marker phyla differentiating the rhizosphere microbial communities associated with the different rootstock types. At the genus level, several taxa belonging to Actinomycetota and Alphaproteobacteria classes were enriched in 1103P genotype rhizosphere. Investigating the potential functional profile, we found that most key enzyme-encoding genes involved in N cycling were significantly more abundant in 5BB rootstock rhizosphere soil. However, we found that 1103P rhizosphere was enriched in genes involved in C cycle and Plant Growth Promotion (PGP) functionality. Our results suggest that the different rootstocks not only recruit specific bacterial communities, but also specific functional traits within the same environment.

## 1 Introduction

Plant–microbe relationships are nowadays considered fundamental to unveil the functioning of the holobiont (Vandenkoornhuyse et al., 2015; Favela et al., 2023). In particular, the “ rhizosphere effect” defined as a cross-talk at the root–soil interface of a plant, which includes root exudation, microbial activity, and nutrient transformation, influences both plant fitness and soil functionality (Haldar and Sengupta, 2015).

In agriculture, the soil microbiome is also now recognized as a component that could influence productivity, adaptation to abiotic stresses (such as those induced by climate change), and prevention/response to pathogenic infections and diseases (Suman et al., 2022; Favela et al., 2023; Nadarajah and Abdul Rahman, 2023). Soil bacteria play an important role in biogeochemical cycles, controlling the availability of essential macro- and micronutrients and modulating plant health (Darriaut, 2022).

The soil microbial composition and function are linked to many driving forces such as soil physico-chemical properties, climate, plant species, and agronomic practices (Hartman et al., 2018; Ren et al., 2018). Independently of environmental factors, primary and secondary metabolites, exuded in a species-specific manner from plant roots, play a key role in the selection of the microbial community (both epiphytic and endophytic) associated with the root system (Pascale et al., 2020; Vanda et al., 2021). Thus, plants can shape the root-associated microbial community by actively and dynamically selecting beneficial bacterial species that support the maintenance of plant fitness and resistance to a particular stress (both biotic and abiotic) (Li et al., 2021).

This is the “cry for help” strategy developed by plant roots (Rizaludin et al., 2021). In viticulture, cultivated grapevines are typically grafted plants composed of a scion cultivar (*Vitis vinifera* L.), which produces grape berries, and a rootstock (*Vitis* sp.), which is selected considering pedoclimatic conditions and to insure tolerance to particular stresses (Wallis et al., 2013), yield, and quality improvement of products (Darriaut et al., 2022b). Plant genetic diversity (both at the scion and the rootstock levels) can influence the microbiota (Bettenfeld et al., 2022). In particular, the grapevine-associated “rhizosphere effect” is mainly modulated by rootstock (acting as the interface with soil) (Liu et al., 2018; Marasco et al., 2018; Bettenfeld et al., 2022), which display a different root system in terms of root architecture and synthesis and exudation of metabolites. In addition, the cultivar also affects this complex and the dynamic process overall, resulting in rhizosphere microbial recruitment. Since the rhizosphere microbiome is now widely recognized as “the emerging barrier in plant–pathogen interactions” (Li et al., 2021), it is therefore necessary to add new knowledge about its biodiversity and functionality and how these can be influenced by agricultural practices. Contrarily to other crops, there is a lack of information on grapevine root-associated microorganisms (Pinto and Gomes, 2016), although it has been recognized that microbial composition and activity are associated with vineyard decline (Darriaut et al., 2021).

While environmental and anthropogenic-driven changes on the grapevine soil microbiome are interesting and widely studied (Steenwerth et al., 2008; Lumini et al., 2009; Schreiner and Mihara, 2009; Marasco et al., 2013; Holland et al., 2014; Vega-Avila et al., 2015; Novello et al., 2017; Berg and Cernava, 2022; Nanetti et al., 2023), the microbial biodiversity dynamics across the rootstock choice is relevant but still little explored (D’Amico et al., 2018; Marasco et al., 2018; Dries et al., 2021; Darriaut et al., 2022a).

However, this information needs to be expanded as it could open up a potentially useful field of research to isolate and promote biofertilizers and bioprotectants. Therefore, the study of plant–microbe interactions in agriculture is potentially useful to improve vine resilience and assist viticulture in an ecological transition of agronomic management that can positively affect both productivity and soil ecological function. This study focused on *Vitis vinifera* L. cultivar Falanghina grafted on PAULSEN 1103 (1103P) and KOBER 5BB (5BB) rootstocks (derived from the breeding of *V. berlandieri* × *V. rupestris* and *V. berlandieri* × *V. riparia*, respectively). We hypothesized that the structure of microbial communities associated with the rhizosphere is driven by rootstock. We further assumed that bacterial functioning was similarly directed by the diversity and structure of the rhizosphere bacterial community. To clarify this hypothesis, we focused on the pathways involved in nutrient cycling (carbon, nitrogen, phosphorous, and sulfur) and plant health. The experimental design of this study aimed to minimize the variability resulting from environmental factors in order to reveal the effect of the rootstock genotype associated with the Falanghina cv. on the bacterial communities in the rhizosphere. This was achieved using plants of the same age, grown on the same type of soil and under the same climatic conditions, and managed with the same agronomic practices. The methodological approach was based on culture-independent approach, based on next-generation sequencing (analysis of the 16S ribosomal RNA), which provides significant advances for exploring the plant microbiome at their natural environment (Pinto and Gomes, 2016). The overall study was aimed at (i) exploring microbial community diversity and structure, (ii) ascertaining the possible influences of rootstock and understanding to which extent rootstocks shape their microbiome, and (iii) revealing the potential function of a microbial community through predictive metagenomics and its shift under plant genotype influence.

## 2 Materials and methods

### 2.1 Experimental design and sampling

**Figure 1:**
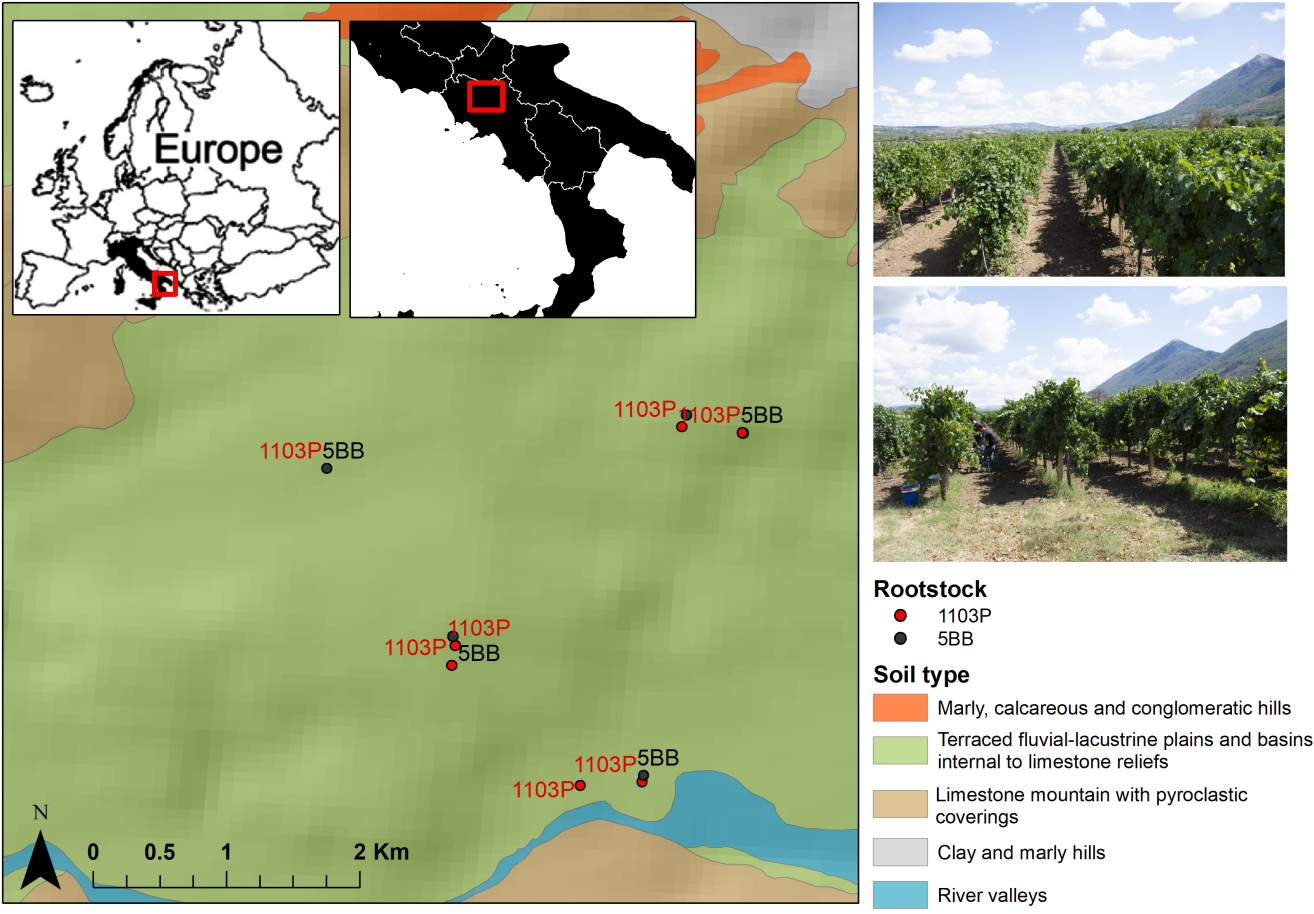
Location map of the study area. Sampling sites of both rootstock (1103P and 5BB) are displayed along with soil subsystems (1:250,000).

Grafted Falanghina grapevine plants (*Vitis vinifera* L. cultivar Falanghina) were sampled in September 2021 (just before the harvest season) at the farming co-operative La Guardiense (Benevento, Sothern Italy), which is one of the biggest in Italy and a representative of the wine-growing region of Sannio. Specifically, two different rootstocks were selected: *V. berlandieri* × *V. rupestris* PAULSEN 1103 (1103P) and *V. berlandieri* × *V. riparia* KOBER 5BB (5BB) (**Table S1** in the Electronic **Supplementary Material**). We investigated seven vineyards subjected to the same pedo-climatic conditions, similar age, training system (double Guyot), spacing, and management (**Figure 1**). Rhizosphere soils were taken from seven 1103P and five 5BB grapevine plants, randomly sampled from three rows of the same vineyard field. Specifically, to sample the rhizospheric soil, three soil cores were taken near the stem of each plant (up to 50 cm). The roots trapped in the soil cores collected near the stem were considered for rhizosphere soil sampling. The three rhizosphere sub-samples were then combined to obtain a homogeneous sample at each plant location. Then, from the three homogeneous samples, a composite one (deriving from the randomly selected plants) was collected. This type of sampling allows us to account for biological variability and reduce sampling bias as much as possible. Overall, 12 rhizosphere soil samples were collected and transported immediately to the Department of Science and Technology (University of Sannio) while maintaining the cold chain. For each sample, 5 g was stored at -80°C for metagenomic analyses.

Additionally, for each vineyard, a composite soil sample was collected from the same three soil cores used for rhizophere soil samples. Specifically, 200 g of soil was stored at -20°C until the agronomic characterization of the soil.

### 2.2 Soil chemical analyses

The physical and chemical properties of the soil were determined at each vineyard (seven) following official analysis methods of the soils (Ministero per le Politiche Agricole e Forestali, 1999): texture, pH, cation exchange capacity (CSC), organic carbon (OC), electrical conductivity (EC), and calcium (Ca), magnesium (Mg), potassium (K), phosphorous (P), and nitrogen (N) content. The soil texture was determined according to Method II (Ministero per le Politiche Agricole e Forestali, 1999). The pH reaction was obtained following the DM 13/09/99 G.U. no. 248 of 21/10/99 Method III.1. The cation exchange capacity (CEC) was obtained according to Method XIII.1 using a molar of ammonium acetate. The exchange bases (calcium, magnesium, potassium, and sodium) removed from the exchange sites with barium chloride solution buffered to pH 8.2 were determined by flame atomization atomic absorption spectrophotometry (FAAS), according to Method XIII.5. The electrical conductivity was determined following Method IV.1 (Ministero per le Politiche Agricole e Forestali, 1999). Total N and P were determined by XIV.3 and XV.1 methods, respectively.

### 2.3 DNA extraction and sequencing

Total genomic DNA was extracted using E.Z.N.A.^®^ Soil DNA Kit (Omega Bio-tek, Inc.) according to the manufacturer’s instructions. Nucleic acid quantification was performed using the Qubit 3.0 fluorometer (Thermo Fisher Scientific, Carlsbad, CA, USA) with Qubit DNA High Sensitivity Assay Kit (Thermo Fisher Scientific). 16S rRNA genes were amplified through PCR using primers for hypervariable regions V2, V3, V4, V6–7, V8, and V9 by using the “Ion 16S Metagenomics Kit” (ThermoFisher Scientific, 5781 Van Allen Way Carlsbad, CA, USA). The libraries were prepared using the Ion Plus Fragment Library Kit (ThermoFisher Scientific) and then sequenced by using Ion 510™ & Ion 520™ & Ion 530™ Kit – Chef on the Ion Torrent ChefTM and the Ion Torrent™ Genestudio S5™ Plus System. We produced around 500,000 reads per sample, on average, by loading 16 samples on 520 ChipTM. The generated unaligned binary data files (Binary Alignment Map, BAM) have been analyzed using Ion Reporter™ Software 5.20 platform with the workflow of Metagenomics 16S w1.1 v5.14. The Ion Reporter workflow is a custom workflow that uses both MicroSEQ™ ID 16S Reference Library v2013.1 and Greengenes v13.5 as reference libraries. All reads shorter than 150 (in base pairs) were filtered out after trimming the primers. A minimum alignment coverage of 90% and a read abundance filter of 10 were set; the threshold value for percentage identity was 99%. 16S rRNA sequence reads were finally clustered into operational taxonomic units (OTUs) using the closed-reference method. The Ion Reporter metagenomics workflow also provides primer information, classification information, percent ID, and mapping information. Concerning the workflow of Metagenomics 16S w1.1 v5.14, a detailed overview is reported in https://assets.thermofisher.com/TFS-Assets/LSG/Vector-Information/ion-reporter-16s-metagenomics-algorithms-whitepaper.pdf.

### 2.4 Data analysis

Soil chemical analyses were presented as mean ± standard deviation; moreover, coefficient of variation (CV) was used to quantify soil feature variations and evaluate the homogeneity of soils.

Microbial community analysis was performed using R v. 3.1.0 (R Core team, 2021) with general dependency on the following packages: *reshape2* (v.1.4.4), *ggplot2* (v.3.4.2), *plyr* (v.1.8.8) (Wickham, 2007; Wickham, 2009; Wickham, 2011) *Hmisc* (v.5.1-0) (Harrell and Dupont, 2019), and *phyloseq* (v.1.42.0) (McMurdie and Holmes, 2013). The 16s data processed into OTUs were imported into R using *phyloseq* to perform exploratory analyses, examining the taxonomic composition and diversity of the samples, and assessing the dissimilarity between groups (5BB and 1103P rootstocks). Specifically, the input data were imported as *phyloseq* object, which is the most commonly used data structure for microbiome data in R environment. For the creation of the *phyloseq* object, the OTU abundance and OTU taxonomy *tsv* files were downloaded from Ion Reporter and subsequently converted into *csv* files, and both were arranged with the additional *csv* file, including metadata, into a combined object.

In particular, *microbiomeMarker*, an R/Bioconductor package (Cao et al., 2022), for microbiome marker identification and visualization was used to process both taxonomic and functional metagenomic data and perform differential analysis (DA).

Liner discriminant analysis effect size (LEfSe) was conducted for metagenomic biomarker discovery after total sum scaling (TSS) data normalization. This was chosen since it addresses the challenge of finding taxa or pathways that consistently explain the differences between two or more microbial communities (in our case study, two different rootstock-associated microbial communities), which is a central problem to the study of metagenomics (Segata et al., 2011). LEfSe was based on wilcoxon_cutoff = 0.05, kw_cutoff = 0.05, and lda_cutoff = 2.

Phylogenetic Investigation of Communities by Reconstruction of Unobserved States (PICRUSt) (Langille et al., 2013) was applied to infer functional categories associated with taxonomic composition (Ijoma et al., 2021). The generated OTU table was also imported into the *PICRUSt* package, and the Kyoto Encyclopedia of Genes and Genomes (KEGG) database was used to predict the functional potential of bacterial communities. The output from the KEGG database containing the predicted function was further analyzed: a single-factor statistical comparison was performed by Mann–Whitney *U* -test to evaluate the functional differences between groups. Then, genes involved in plant growth promotion (PGP) and encoding key enzymes in nutrient cycling were accordingly identified in the resulting profiles using their KEGG orthologs (Sashidhar and Podile, 2010; Kaiser et al., 2016; Marasco et al., 2018) and plotted using *ggplo2* package.

## 3 Results

### 3.1 Soil features

The soil information were prior retained in the information system managed by the Agriculture Research Council of Italy (Costantini et al., 2014). The available soil subsystems geodatabase (1:250,000) was considered. The sampled vineyard soils are grafted onto flood plains and terraces and were classified as Calciustepts and Haplustepts according to the Keys to Soil Taxonomy (Soil Survey Staff, 2015). All soil samples were sandy clay loam and silty clay loam (**Table 1**). Based on our analyses, the soil pH was 8.02, on average, typical of alkaline soils, with a low CSC (<10). The organic carbon content ranged from low to normal, with an average value of 11.28 g/kg, and was comparable to data reported for European Mediterranean cropland semiarid soils (Tóth et al., 2013; Grilli et al., 2021). The total N mean value was 0.76 g/kg. To exclude biases due to different soil features, which play a key role in shaping the microbiome layout, the variability of soil characteristics was inspected by coefficient of variation (CV, %), which is a useful statistical parameter for comparing the degree of variation of the samples. The CV data (**Table 1**) showed that the soil samples were quite homogenous as spread around, and the mean is overall less than 20% (Selmy et al., 2022). However, the statistical analysis demonstrated a high variability of EC with a coefficient of variation of 62.5%. The soil pH and CEC levels showed minimum variability in comparison to other soil properties.

**Table 1:** Soil features of soil from vineyards in the 0– 50-cm soil layer; mean and standard deviation (N = 7) are shown.

Parameter	Measure unit	Value (mean ± standard deviation)	Coefficient of variation (CV), %
Texture	–	sandy clay loam - silty clay loam	
pH	Unit	8.02 ± 0.13	1.61
CEC	meq/100g	9.17 ± 1.35	14.7
OC	g/kg	11.28 ± 2.38	29.9
EC	dS/m	1.44 ± 0.7	62.5
K	meq/100g	0.95 ± 0.24	24.8
Mg	meq/100g	3.57 ± 1.14	31.9
Ca	meq/100g	2.32 ± 0.44	19.1
P	mg/kg	18.24 ± 2.87	15.7
N	g/kg	0.76 ± 0.15	19.4

### 3.2 Overall structure of bacterial communities associated with the root system

**Figure 2:**
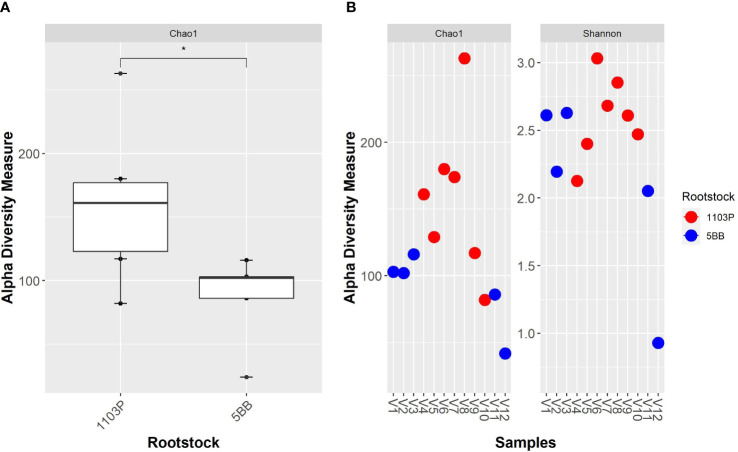
Alpha - diversity measure of grapevine rhizosphere bacterial microbiota from different rootstocks (5BB and 1103P). **(A)** Boxplot of Chao1 index; comparisons between rootstocks were performed by the Wilcoxon test (* indicate significance at *p* < 0.05). **(B)** Chao1 and Shannon indexes of each sample.

A total of 4,293,111 amplicon sequences from 12 samples were collected (**Table S2** in the Electronic **Supplementary Material**). The bacterial community dataset included 883,864 quality-filtered reads, with 18,988 to 130,421 reads per sample (**Table S2**). We identified 458 taxa based on the conventional criterion of 99% sequence similarity. Alpha-diversity measures (**Figure 2**) indicate that the two rootstocks’ rhizosphere microbiota are significantly different (*p* < 0.05). Overall, the bacterial alpha-diversity (observed OTUs) significantly increased in the 1103P rhizosphere microbiota. Interestingly, the 5BB soil microbiome showed the lowest within-group variability, while greater dispersion was observed for the rhizosphere microbiome of 1103P. The Shannon diversity measure (**Figure S1** in the Electronic **Supplementary Material**) indicates no significant difference between groups.

**Figure 3:**
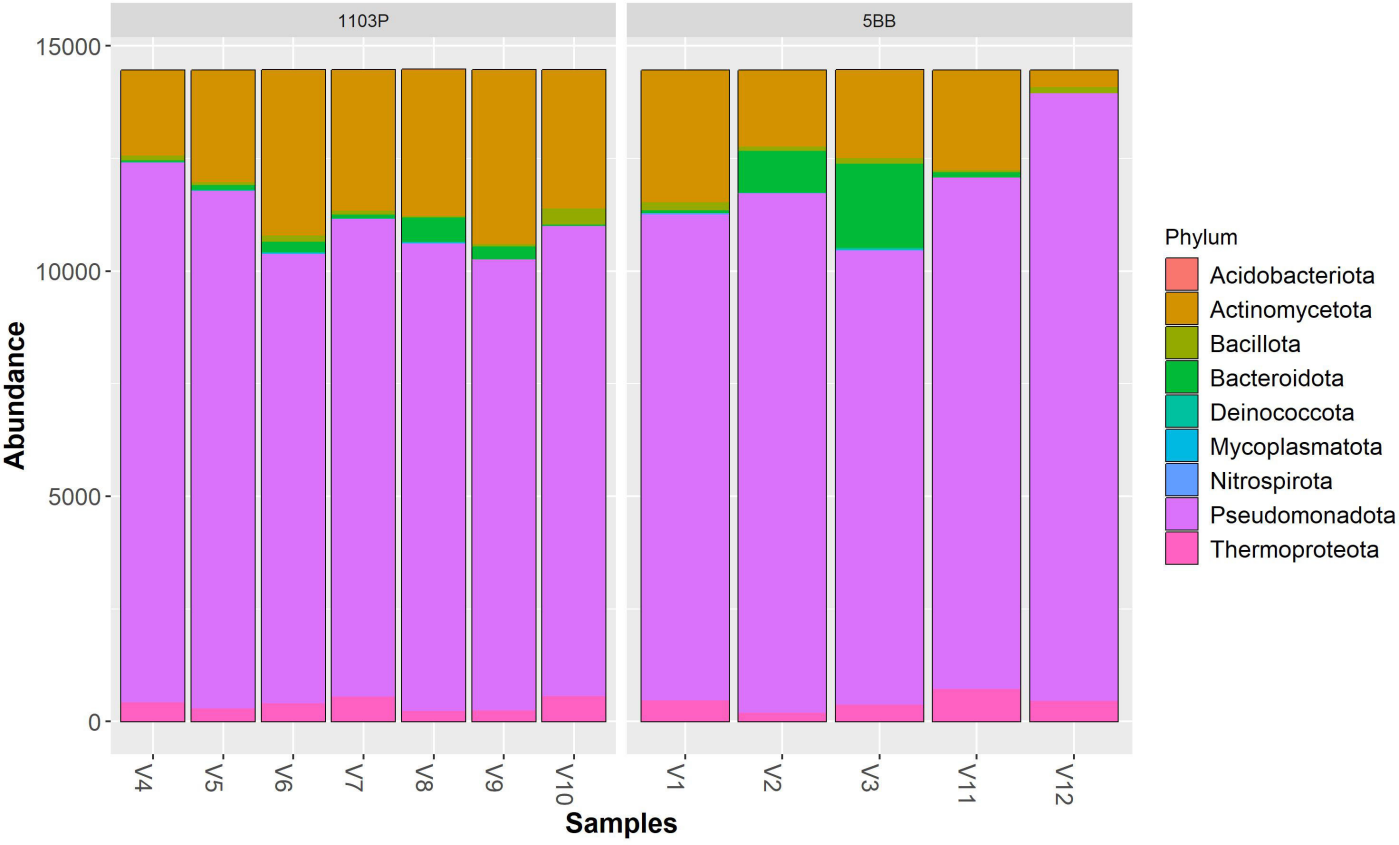
Bar plot of reads abundance in each sample using a *phyloseq* package. The reads are ordered by phylum; the samples are divided by rootstock type (5BB and 1103P).

**Figure 4:**
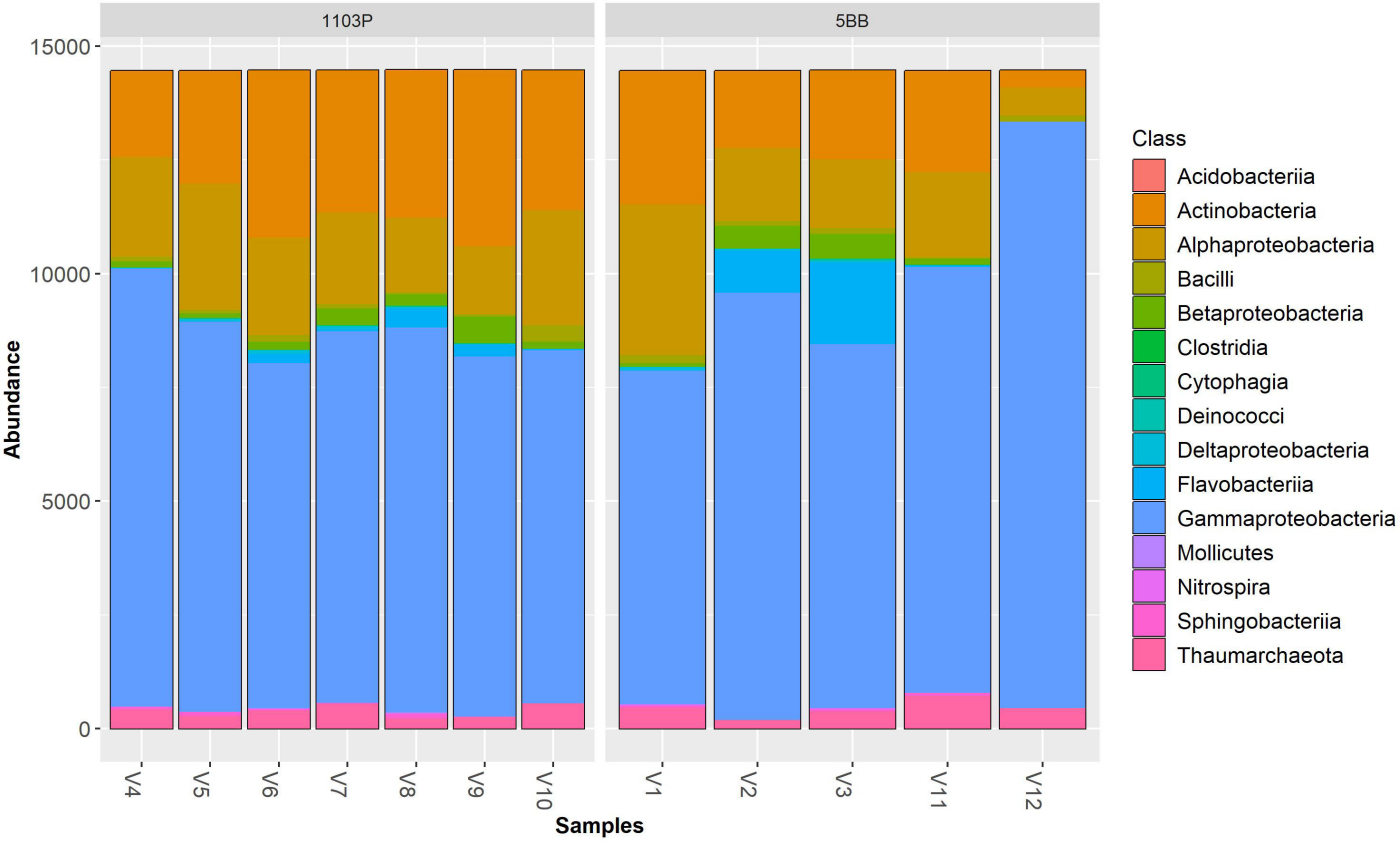
Bar plot of reads abundance in each sample using a *phyloseq* package. The reads are ordered by class; samples are divided by rootstock type (5BB and 1103P).

Irrespective of the cultivar, Pseudomonadota (synonym Proteobacteria) was the dominant phylum (**Figure 3**), followed by Actinomycetota (synonym Actinobacteria) > Bacteroidota (synonym Bacteroidetes) > Thermoproteota (synonym Crenarchaeota). Pseudomonadota ranged from 69.19% (V9 sample) to 93.34% (V12 sample). Actinomycetota ranged from 2.56% (V12 sample) to 26.71% (V9 sample). At the class level (**Figure 4**), the most abundant taxa in all samples were Gammaproteobacteria (64.92% and 57.37%, on average, in the 5BB and 1103P rhizosphere samples, respectively) > Actinobacteria (12.70% and 21.08%, on average, in the 5BB and 1103P rhizosphere samples, respectively) > Alphaproteobacteria (12.37% and 14.62%, on average, in the 5BB and 1103P rhizosphere samples, respectively).

At the family level (**Figure S2** in the Electronic **Supplementary Material**), Pseudomonadaceae was the most abundant taxa (with a relative abundance ranging from 48.61% to 88.07%) in all samples. The second most representative family were Rhodospirillaceae (V1, V10, V4, and V5 samples), Nitrososphaeraceae (V11 and V12 samples), Flavobacteriaceae (V2 and V3 samples), Nocardioidaceae (V6, V7, and V8 samples), and Micrococcaceae (V9 samples).

### 3.3 Differential OTU distribution and functional profile

In order to obtain further information on the recruitment of the microbiome that thrives in the rhizosphere of the two different rootstocks, a DA was performed.

**Figure 5:**
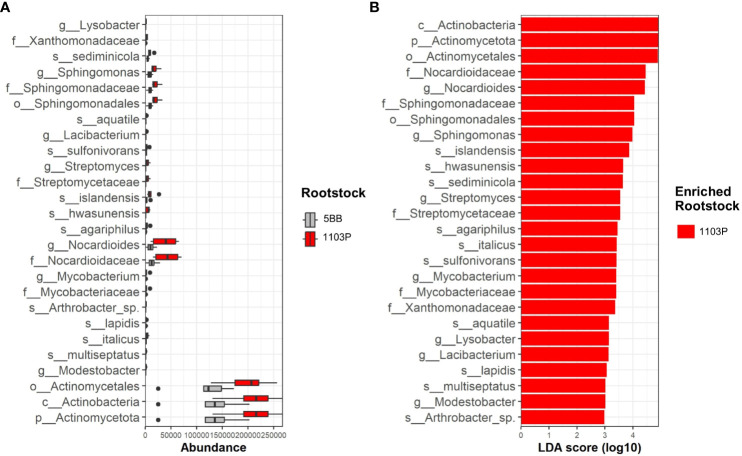
Rhizosphere microbiome distribution between 5BB and 1103P rootstock. **(A)** Box plots showing the interquartile range of median abundance of statistically significant comparisons between 5BB (orange) and 1103P (blue) using multiple corrections-adjusted Kruskal–Wallis tests (*p* < 0.05) at the family level. The line in the center shows the median. **(B)** LEfSe bar chart showing the taxa that are enriched in the two different rhizosphere-associated rootstocks; the LDA scores represent the effect size of each abundant species. Species enriched in each group with an LDA score > 2 were considered (LDA score threshold = 3.5).

The LEfS robustly identified rhizosphere microbial taxa that are statistically different among the two investigated rootstocks. The LEfSe detected 26 differentially abundant taxa in the rhizospheres (**Figure 5**), which discriminated the bacterial communities between the different root genotypes.

**Figure 6:**
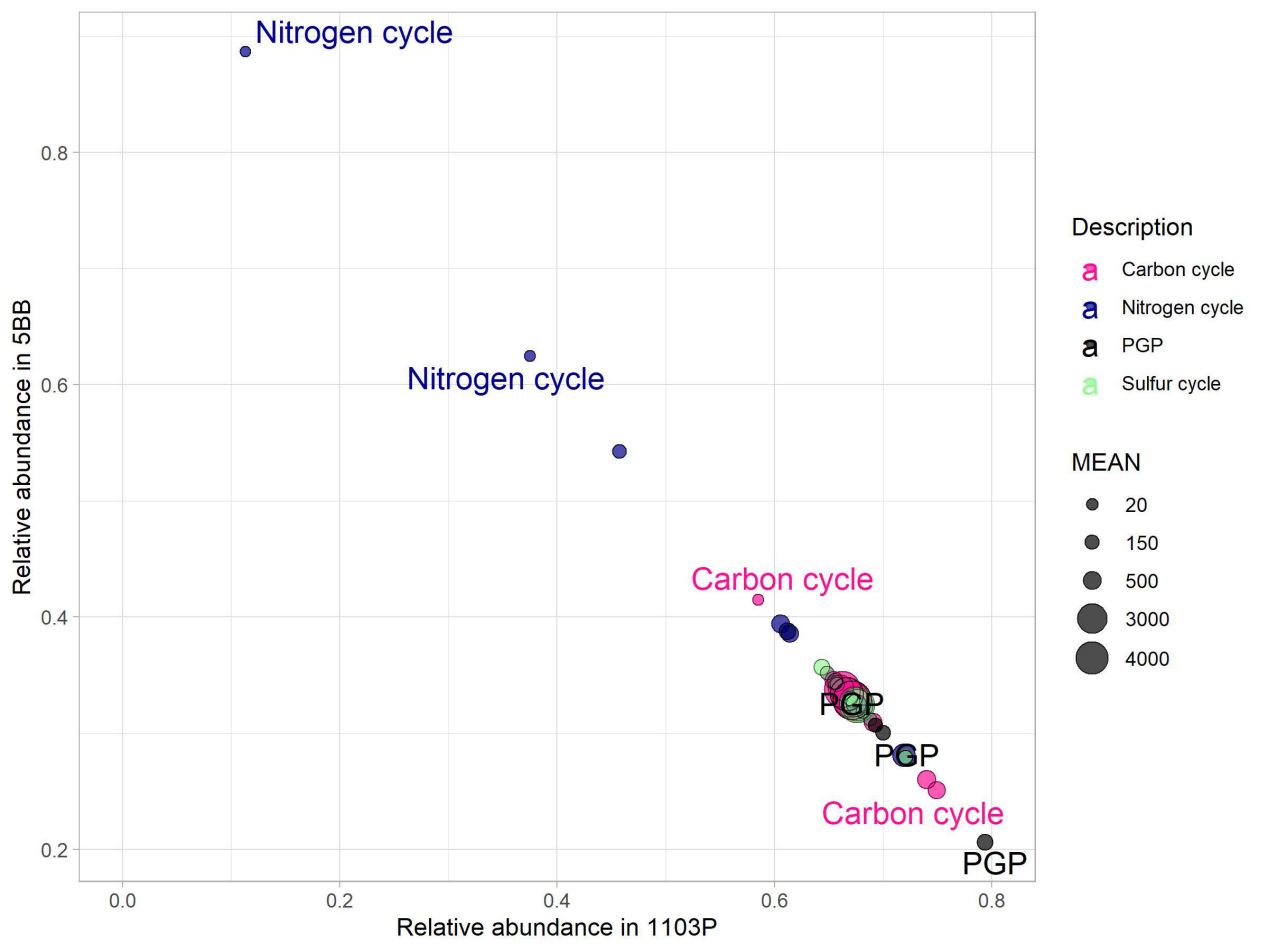
Key genes (KO) for carbon, nitrogen, and sulfur cycles and PGP activities. Their mean abundance (relative to the mean in the complete dataset) in the 5BB rhizosphere soil was plotted against the mean abundance in the 1103P rhizosphere soil. The size of the circles indicate the mean abundance in the complete dataset. The labeled features indicate a significantly different KO (*p* < 0.05, Mann–Whitney *U*-test).

Actinomycetota was the major phyla that contributed to differentiate the rhizosphere microbial communities associated with the different rootstock types (**Figure 5**). In particular, at the genus level, g:Nocardioides contributed to the relative high dissimilarity between 5BB and 1103P (enriched in the latter) with a LDA score above 4.5. Among Actinomycetota, the genus g:Mycobacterium, g:Agromyces, g:Streptomyces, g:Modestobacter, and g:Arthrobacter also determined the dissimilarities among rootstocks in the rhizosphere fractions, being enriched in 1103P (with a LDA score ranging from 3 to 3.6). We also found different microbial biomarkers belonging to Alphaproteobacteria class, such as the genus g:Sphingomonas, g:Hyphomicrobium, and g:Lacibacterium. Finally, among Gammaproteobacteria, the 1103P rhizosphere microbiome revealed that the genus g:Lysobacter was also enriched with respect to 5BB.

prediction of functional content of microbiome from the rhizosphere of both rootstocks was obtained by processing the OTU table on PICRUSt using the KEGG database. We found 2,179 molecular functions in terms of functional KEGG orthologs (KO) in our samples. However, we focused on the pathways involved in carbon, nitrogen, and phosphorus cycling and PGP activities (**Figure 6**) and found 40 KO to be involved. A total of 15 KO were associated to C cycle, 12 to N cycle, three to P cycle, and four to S cycle. In addition, we also found five KO to be associated to PGP activities. Comparing the soil microbiomes associated with the two rootstocks by Mann–Whitney *U*-test (*p* < 0.1), we found a significant effect of plant genotype.

Most key enzyme-encoding genes involved in the cycling of N were more abundant in the 5BB rootstock rhizosphere soil (Mann–Whitney test, *P* < 0.1; **Figure 6**). Specifically, these were involved in dissimilatory nitrate reduction (KO00362) and Anammox (KO10535) pathways and were 1.7- and 7. 8-fold higher in the functional profile associated to 5BB.

As regards the C cycle, chitinase genes (involved in the chitin breakdown pathway) showed a 1.4-fold higher abundance in the 1103P-associated rhizobiome compared to 5BB. In addition, 1103P also had a 2.9-fold higher abundance of carbon monoxide dehydrogenase (K00192). Interestingly, we found that the 1103P rhizosphere was also significantly enriched in microbes with a PGP functionality compared to 5BB. Specifically, for potential PGP traits involved in plant drought tolerance (K01575) (Vigani et al., 2019), a 3.8-fold higher abundance in 1103P was observed. Genes that encode enzymes linked to either nitrilase route in indole acetic acid (IAA) biosynthesis (K01501) and 1-aminocyclopropane-1-carboxylate (ACC) deaminase activity (K01505) were enriched in 1103P (2.3- and 2. 2-fold, respectively).

However, considering the PGP key enzyme-encoding genes for biostimulation, IAA production (K01426), and auxin production (K00817), no rootstock effect was identified. In addition, no differences of functionality related to the S cycle were observed.

## 4 Discussions

To our knowledge, this work is the first to study the compositional structure of the rhizosphere soil microbiota from *V. vinifera* cultivar Falaghina grafted on two different rootstock hybrids: PAULSEN 1103 (1103P) and KOBER 5BB (5BB).

To observe the contribution of the genotype in shaping the soil microbial communities associated with the rhizosphere, we sampled rhizosphere soil from vineyards grafted onto the same soil type, under the same bio-geographical factors, and with the same type of management. Soil chemical analyses confirmed that they are, overall, not very heterogeneous at this scale. By means of next-generation sequencing of the 16S rRNA gene, we obtained a metagenomic profile of the investigated rhizospheres. The obtained data showed that the bacterial communities associated with each rootstock type substantially differed in richness (Chao1 index, **Figure 2**), indicating a significantly higher number of bacterial taxa in the rhizosphere of 1103P [in contrast to the study of D’Amico et al. (2018)], which found an opposite behavior for Barbera cultivar). However, although the Shannon index was also higher (on average) in the 1103P samples, the magnitude of inflation was much smaller than that of the species richness estimators (Chao1), and no significant differences were observed between the two types of samples (**Figure S1**). The motivation for this result is that the Shannon diversity index is based on evenness and is more dependent on highly abundant OTUs rather than species richness estimates (Wang et al., 2012). Irrespective of the cultivar, Pseudomonadota and Actinomycetota were the dominant phyla, as also observed in other researches on *Vitis vinifera* rhizosphere (e.g., Marasco et al., 2018). At the family level, we found that Pseudomonadaceae was the most abundant taxa. This family includes several genera (including *Pseudomonas*) known to promote plant growth and protect plants from pathogen attack (Raaijmakers et al., 2009; Mendes et al., 2013). Worthy of note is the absence of Agrobacterium taxa (Armijo et al., 2016), which are known to induce grapevine crown gall development (*A. vitis*), in all the analyzed rhizospheres.

The LEfSe revealed that the rootstock type had a significant influence on the microbial populations colonizing the rhizosphere and highlighted several marker genes of the 1103P rhizosphere- associated microbiome. Among these, we observed the presence of several Actinobacteria classes. The data also revealed different microbial biomarkers belonging to Alphaproteobacteria and Gammaproteobacteria. It is interesting to note that the genus *Sphingomonas* is a biomarker of 1103P because, in addition to having a role in solubilizing K (Zhang and Kong, 2014), recent studies have shown that endophytic *Sphingomonas* have a role in promoting plant growth by increasing gibberellins, IAA (Khan et al., 2014), and glutathione biosynthesis (Pan et al., 2016). In addition, the *Hyphomicrobium* genus, known for PGP activities (Rani et al., 2021), characterized the 1103P rhizosphere.

Therefore, it was not surprising that the analysis of the potential functionality of the microbiome (obtained through PICRUST bioinformatic analyses) showed a significantly higher potential for PGP activity in the rhizosphere of the 1103P rootstock (**Figure 6**). Moreover, also concerning KO involved in the C cycle, we found a significantly marked potential in 1103P. This might result from the higher abundance of Actinomycetota taxa in the 1103P soil, as this group is known to harbor a high number of genes involved in the C cycle [as also reported in Bai et al. (2016) and Kaiser et al. (2016)]. Interestingly, the microbial potential function related to the N cycle was higher in the 5BB rootstock. Although our analyses investigated just the potential functional profile, the obtained results suggest that the different rootstocks select not only for distinct bacterial taxa but also for specific functional traits within their bacterial communities. In this study, the physico-chemical properties of the soil, local biogeographical factors, and vineyard management procedures (which influence the composition of root communities throughout the vine life cycle) can be considered quite uniform for all the rootstocks sampled and analyzed. Consequently, it is reasonable to assume that the variability in the structure and potential functions of the rhizosphere bacterial communities can be attributed to the rootstock genotype (which is a driver of the plant-associated microbiota), through (i) different colonization of the root system, (ii) variations in the physiology and growth of the plant, (iii) root exudation, and (iv) random forces (DeAngelis et al., 2009; Marasco et al., 2018).

The plant host and associated microorganisms are involved in a proper cross-talk aimed at defining a stable holobiont in which the partners co-exist, communicate, and cooperate for the fitness of the whole organism (Berlanga-Clavero et al., 2020).

Since the diversification of the root microbiota (capable of generating the endophytic microbial community) begins in the rhizosphere fraction (Bulgarelli et al., 2013; Samad et al., 2017), it is crucial to add new knowledge to the specific influence of plant genotype in the modulation of the root microbiome. Understanding rootstock microbial recruitment can provide not only useful information to understand the microbial *terroir* (Gilbert et al., 2014) but also new insights into vineyard management and the plant’s response to both biotic and abiotic stress factors, which represent an increasingly important challenge for viticulture.

Following this first study, the future aims are to (i) provide information on the fungal diversity of the associated rhizosphere, (ii) understand, through a larger and more detailed experimental design, how management may influence the structure of the rhizosphere microbiome and the co-occurrence of bacterial and fungal taxa as well as their function, and (iii) address the functional features of the microbiota through transcriptomic approaches.

## Data availability statement

The data presented in the study are deposited in the SRA repository, accession number PRJNA956887.

## Author contributions

DZ: Conceptualization, Data curation, Formal analysis, Investigation, Supervision, Writing – original draft; CG: Conceptualization, Funding acquisition, Resources; RS; Funding acquisition, Resources; GM: Funding acquisition; MT: Investigation, Data curation; AP: Investigation, Data curation; AF: Investigation; MM: Investigation; MAR: Investigation. All authors contributed to Manuscript Revision.
