# Supplementary material for: The rootstock shape microbial diversity and functionality in the rhizosphere of Vitis vinifera L. cultivar Falanghina
Source: Front Plant Sci. 2023 Aug 14;14:1205451. doi: 10.3389/fpls.2023.1205451 (PMC10461393; doi:10.3389/fpls.2023.1205451)

**Supplementary Material**

### The rootstock shape microbial diversity and functionality in rhizosphere of *Vitis vinifera* L. Cultivar Falanghina

**Daniela Zuzolo^1^, Maria Antonietta Ranauda^1^, Maria Maisto^1^, Maria Tartaglia^1*^, Antonello Prigioniero^1^, Alessandra Falzarano^1^, Giuseppe Marotta^2^, Rosaria Sciarrillo^1^, Carmine Guarino^1^**

^1^Department of Science and Technologies, University of Sannio, Benevento, Italy

^2^Department of Law, Economics, Management and Quantitative Methods (DEMM)

*** Correspondence:**

[mtartaglia@unisannio.it](mailto:mtartaglia@unisannio.it)

Table S1 – Sampled sites (vineyards)

| **Sampling sites** | **Rootstock** |
| --- | --- |
| V6 | 1103P |
| V7 | 1103P |
| V8 | 1103P |
| V9 | 1103P |
| V10 | 1103P |
| V11 | 5BB |
| V12 | 5BB |
| V1 | 5BB |
| V2 | 5BB |
| V3 | 5BB |
| V4 | 1103P |
| V5 | 1103P |

Table S2 – Amplicon sequences collected from rhizosphere soil samples.

| **Sample** | **Total number of reads** | **Number of valid reads** | **Number of reads ignored (due to low number of copies <10)** | **Mapped reads in sample** | **Un-Mapped reads in sample** |
| --- | --- | --- | --- | --- | --- |
| V1 | 428373 | 428373 | 219522 | 94506 | 3076 |
| V2 | 491358 | 358259 | 251537 | 96038 | 10684 |
| V3 | 567333 | 416999 | 416999 | 129912 | 13160 |
| V4 | 233565 | 174337 | 126684 | 43216 | 4437 |
| V5 | 491358 | 358259 | 251537 | 96038 | 10684 |
| V6 | 244643 | 178070 | 128420 | 47842 | 1808 |
| V7 | 117815 | 86944 | 65226 | 18988 | 2730 |
| G1 | 269271 | 191975 | 139337 | 50501 | 2137 |
| G2 | 237420 | 176068 | 127455 | 42865 | 5748 |
| G3 | 274128 | 203262 | 147987 | 49221 | 6054 |
| G4 | 577831 | 419686 | 270972 | 130421 | 18293 |
| G5 | 360016 | 269060 | 182306 | 84316 | 2438 |
| Total (12 samples) | 4293111 | 3261292 | 416999 | 883864 | 81249 |

Figure S1 –Boxplot of diversity measure (Shannon index) of grapevine rhizosphere bacterial microbiota from different rootstock (5BB and 1103P); comparisons between rootstocks was performed by Wilcoxon test (* indicate significance at *p* < 0.05, ns indicate no significance).


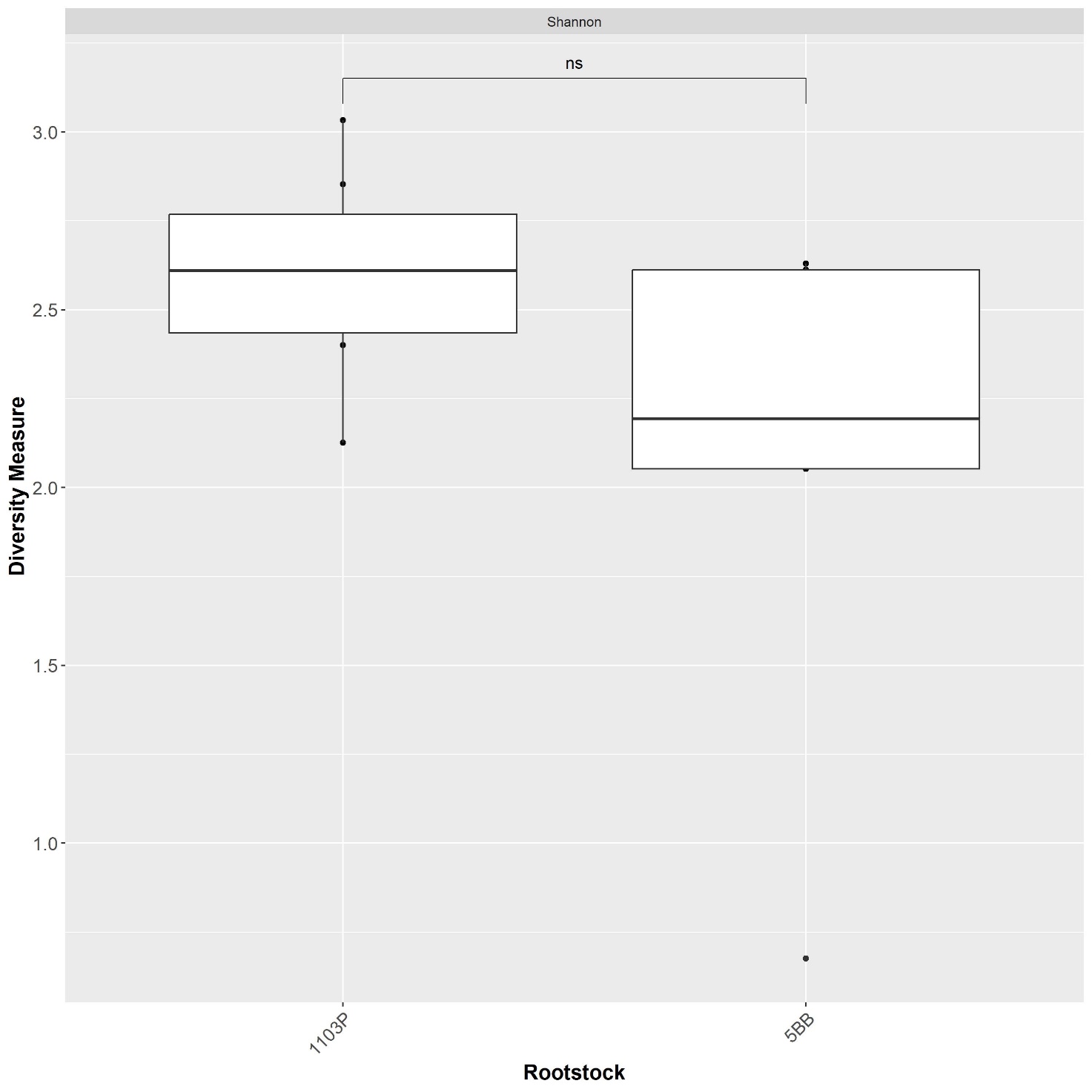


Figure S2 - Bar plot of reads abundance in each sample using a *phyloseq* package. The reads are ordered by family; samples are divided by rootstock type (5BB and 1103P).


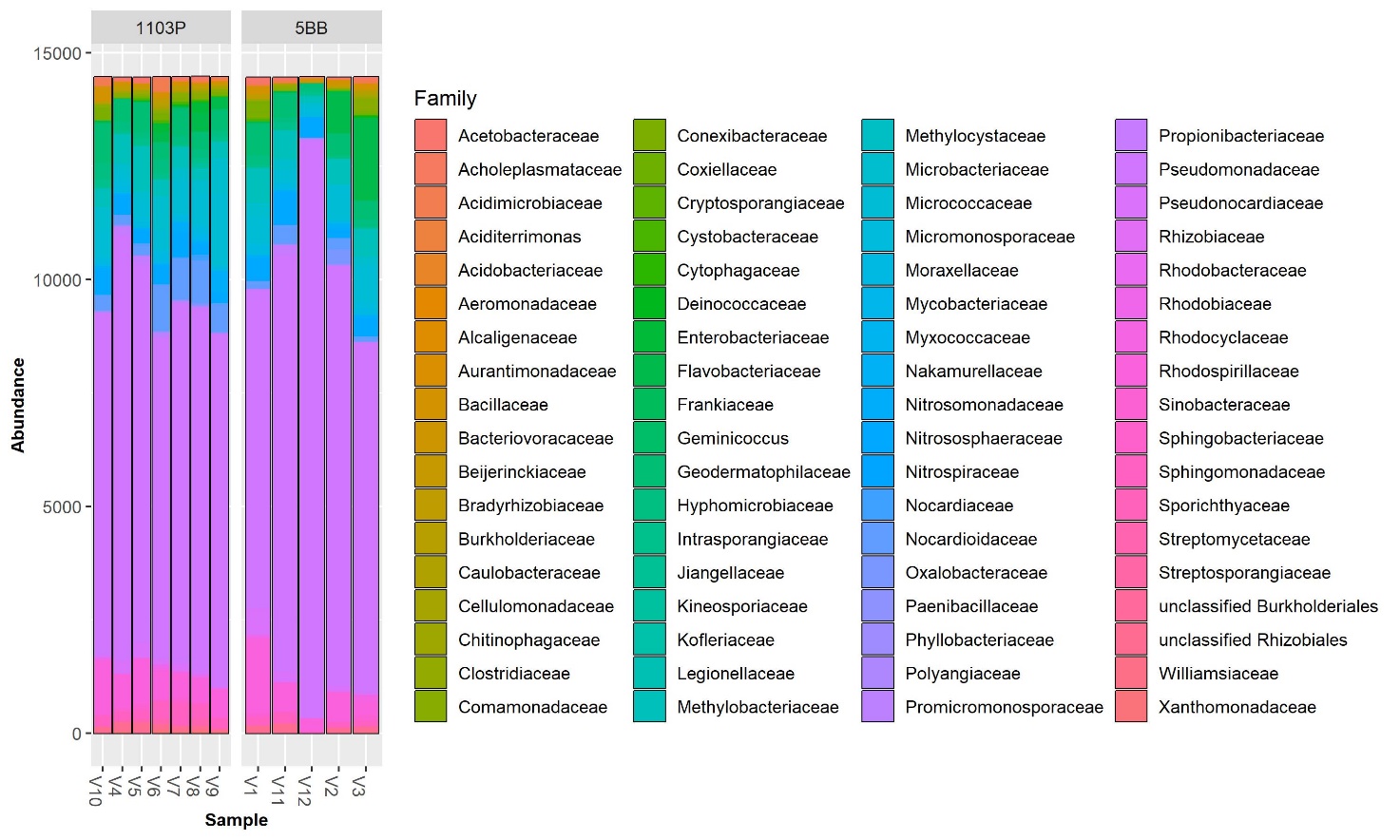

Supplement: Supplementary file 1 [file DataSheet_1.docx]
